# Protective effect of corn peptides against alcoholic liver injury in men with chronic alcohol consumption: a randomized double-blind placebo-controlled study

**DOI:** 10.1186/1476-511X-13-192

**Published:** 2014-12-13

**Authors:** Yuhong Wu, Xingchang Pan, Shixiu Zhang, Wenxian Wang, Muyi Cai, Yanrong Li, Fan Yang, Hongwei Guo

**Affiliations:** Department of Nutrition and Food Hygiene, School of Public Health, and Key Laboratory of Public Health Safety (Ministry of Education), Fudan University, Shanghai, 200032 China; School of Medicine, Hangzhou Normal University, Hangzhou, 310036 Zhejiang China; China National Research Institute of Food and Fermentation Industries, Beijing, 100027 China; Institute of Nutrition and Food Hygiene, School of Public Health, Shandong University, Jinan, 250012 China

**Keywords:** Corn peptides, Hepatoprotective, Oxidative stress, Lipid profile, Alcoholic liver injury, Human study

## Abstract

**Background:**

Corn peptides (CPs) are a novel food prepared from corn gluten meal, which is a main by-product of the corn starch industry. Recently, significant beneficial effects of CPs on early alcoholic liver injury in rats and on acute alcoholic injury in mice were observed. To our knowledge, the present study is the first report showing that CPs supplementation has beneficial effects on lipid profile, oxidative stress and alcoholic liver injury in men with chronic alcohol consumption.

**Methods:**

A 9-week, randomized, double-blind, placebo-controlled study was conducted between September 2011 and August 2012 to assess the hepatoprotective effect of CPs. A total of 161 men were randomized to receive CPs (*n* = 53), whey protein (*n* = 54), or corn starch placebo (*n* = 54) at the same dose of 2 g twice daily. 146 participants completed the study. Serum lipid profile, serum markers of liver injury, oxidative stress and inflammation, and fatty liver based on the results of abdominal ultrasonography were assessed at the beginning and end of the intervention.

**Results:**

CPs supplementation (4 g/d) for 9 weeks significantly lowered serum levels or activities of total cholesterol, triglyceride, alanine aminotransferase, aspartate aminotransferase, malondialdehyde and tumor necrosis factor-α, and significantly increased serum activities of superoxide dismutase and glutathione peroxidase, but the same dose of whey protein and corn starch (placebo) did not demonstrate these effects.

**Conclusions:**

Our results indicate that CPs may have protective effects on alcohol-induced liver damage via modulation of lipid metabolism and oxidative stress. CPs may potentially be used as a functional food for the management of alcoholic liver disease in subjects with chronic alcohol consumption.

**Electronic supplementary material:**

The online version of this article (doi:10.1186/1476-511X-13-192) contains supplementary material, which is available to authorized users.

## Background

It is estimated that almost 4% of all deaths worldwide are attributable to alcohol, 6.2% for men and 1.1% for women. The harmful use of alcohol is the leading risk factor for death in men aged 15–59 [1]. Hepatic steatosis is the initial stage of alcoholic liver injury, and it can progress to steatohepatitis, fibrosis, and cirrhosis, which is the third leading (at 16.6%) cause of alcohol-attributable deaths worldwide [1]. A comprehensive understanding of alcoholic liver disease (ALD) mechanisms is incomplete, but increasing evidence supports the notion that oxidant stress plays a crucial role in the progression of ALD to cirrhosis [2, 3]. Notably, numerous antioxidants have been shown to protect against the damaging effects of alcohol in *in vitro* and *in vivo* models of ALD [2, 4, 5]. Therefore, although abstinence from alcohol is the cornerstone and long-term goal for the management of all stages of ALD, the research and development of safe dietary supplements, such as antioxidants of food origin, to alleviate alcoholic liver injury is an attractive and important challenge for the scientific community.

Corn gluten meal (CGM) is a main by-product of the corn starch industry. More than 840 000 tons of CGM are produced in China annually. However, CGM is mainly used as a feedstuff or discarded because of its low water solubility and severe amino acid imbalance even though it contains approximately 60% (w/w) protein [6, 7]. In recent years, corn peptides (CPs), a novel food prepared from CGM by enzymatic hydrolysis, have attracted considerable interest due to various bioactive properties, including antioxidant activity [6, 8–14], improvements in lipid profiles [9] and the ability to accelerate alcohol metabolism [15, 16] and protect against alcohol-induced liver injury [9, 10]. The research and development of CPs might effectively increase the value of CGM in the marketplace. However, most studies using CPs have been conducted in animals or *in vitro*. Human data on the effect of CPs are limited. Our previous work showed that CPs supplementation accelerated alcohol clearance (based on blood alcohol concentration and breath alcohol concentration) in healthy young men, especially at a dose of 4 g/d (XP,YW, FL, SZ, FY, YL, MC and HG, unpublished data). This study assessed the effect of CPs (4 g/d) on lipid profile, oxidant stress, inflammation and alcoholic liver injury in men with chronic alcohol consumption and fatty liver.

In addition, convincing evidence indicates that whey protein (WP), which is one of the most popular protein supplements on the markets today, may also have beneficial health effects of antioxidant [17–19], anti-inflammatory [19], lipid metabolism improvements [19] and hepatoprotection [17, 20] including protection against nonalcoholic fatty liver [21, 22]. The amounts of WP administered to adult humans in most previous studies have been 20–60 g [19]. However, WP in the current study was given orally to subjects (WP group) in the same dose regimen as CPs (4 g/d) to compare the effect of the peptide and protein.

Therefore, the present study evaluated the effects of CPs supplementation on lipid profile, oxidative stress and alcoholic liver injury compared to WP and corn starch (placebo) supplementation in men with chronic alcohol consumption. In addition to serum markers, ultrasound examination was performed before and after the interventions to explore the effect of supplements on fatty liver.

## Results

### Participant characteristics and compliance

**Figure 1:**
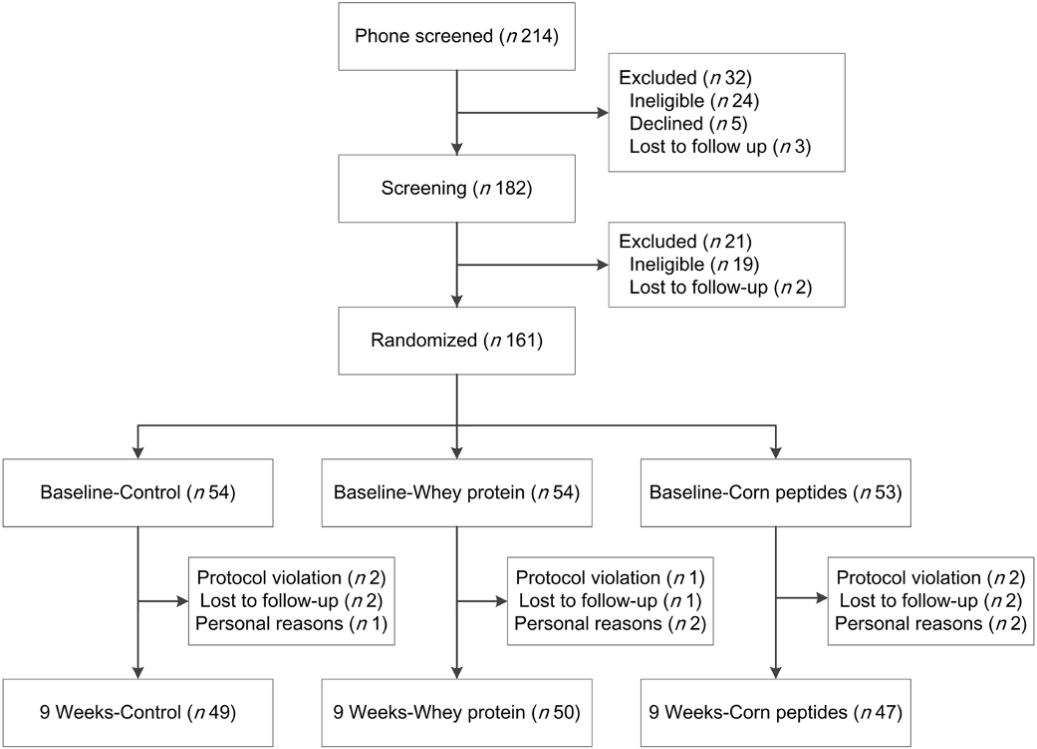
**Study flow design.**

Participant flow and follow-up are given in Figure 1. Of the 161 participants recruited into the study, 15 withdrew from the study (9.3%): placebo group (*n* = 5, 9.3%), WP group (*n* = 4, 7.4%) and CPs group (*n* = 6, 11.3%) (χ^2^ = 0.485, *P* = 0.785). Data from the 146 participants who completed the study were included in subsequent analyses. Self-reported data from the logs and returned supplements indicated that participants complied very well throughout the study. Overall, 90% of the participants consumed >90.0% of the provided supplements (see Additional file 1: Table S1). Compliance did not differ between treatment groups (*P* = 0.767). No associated adverse events were reported during this study. Participant characteristics are given in Table 1. Demographic and anthropometric characteristics of participants did not differ significantly between groups.

**Table 1:** **Baseline characteristics of the subjects**

Parameters	Placebo (***n*** = 49)	Whey protein (***n*** = 50)	Corn peptides (***n*** = 47)
Age (years)			
Median	47.0 (39.5 - 52.0)	49.0 (43.8 - 52.0)	45.0 (38.0 - 52.0)
Weight (kg)	76.8 ± 0.7	77.2 ± 0.8	78.0 ± 0.9
BMI (kg/m^2^)	25.9 ± 0.2	26.2 ± 0.2	26.4 ± 0.3
>high school diploma (%)	21 (42.9)	16 (32.0)	18 (38.3)
>RMB 40,000 yearly income (%)	19 (38.8)	18 (36.0)	23 (48.9)
Cigarette smoker (%)	23 (46.9)	30 (60.0)	26 (55.3)
Alcohol consumption (g/day)	44.3 (36.0-39.6)	45.6 (36.0-60.0)	46.9 (36.0-48.0)
Duration of consumption (%)			
5–10 years	8 (16.3)	9 (18.0)	4 (8.5)
10–20 years	17 (34.7)	16 (32.0)	16 (34.0)
>20 years	24 (49.0)	25 (50.0)	27 (57.5)

BMI, body mass index.

Values are means ± SE (continuous variables, normal distribution), medians with interquartile ranges (continuous variables, skewed distribution) in parentheses or numbers with percentages in parentheses (categorical variables). Continuous variables were compared using one-way ANOVA or Kruskal-Wallis H test, and categorical variables were compared using chi-square test or Kruskal-Wallis H test. Characteristics did not differ between groups.

### Serum lipid profile

The effects of WP and CPs on lipid profiles are given in Table 2. At week 9, after adjusting for baseline covariates, the CPs group had significantly greater reductions in serum concentrations of triglyceride (TG) (*P* < 0.001 and =0.003, respectively) and total cholesterol (TC) (*P* = 0.007 and 0.035, respectively) from baseline values compared to the placebo and WP groups. Changes in low-density lipoprotein cholesterol (LDL-C) and high-density lipoprotein cholesterol (HDL-C) did not differ between groups.

**Table 2:** **Effects of treatments on lipid profile**

Parameters	Placebo (***n =*** 49)	Whey protein (***n =*** 50)	Corn peptides (***n =*** 47)	***P***value
TG (mmol/L)				
Baseline	2.53 (1.84 - 3.68)	2.41 (1.56 - 3.71)	2.61 (1.84 - 4.13)	0.474^A^
∆9 weeks	-0.13 ± 0.07^a^	-0.25 ± 0.07^a^	-0.57 ± 0.08^b^	<0.001^B^
TC (mmol/L)				
Baseline	5.23 ± 0.13	5.13 ± 0.14	5.42 ± 0.17	0.372^A^
∆9 weeks	-0.02 ± 0.10^a^	-0.11 ± 0.10^a^	-0.42 ± 0.11^b^	0.020^B^
LDL-C (mmol/L)^C^				
Baseline	2.69 ± 0.12	2.90 ± 0.15	2.91 ± 0.18	0.497^A^
∆9 weeks	0.05 ± 0.12	-0.10 ± 0.11	-0.23 ± 0.12	0.247^B^
HDL-C (mmol/L)				
Baseline	1.26 ± 0.03	1.27 ± 0.03	1.24 ± 0.03	0.740^A^
∆9 weeks	0.02 ± 0.02	0.04 ± 0.02	0.08 ± 0.02	0.055^B^

TG, triglyceride; TC, total cholesterol; LDL-C, low-density lipoprotein cholesterol; HDL-C, high-density lipoprotein cholesterol.

Values are means ± SE (baseline, normal distribution), medians with interquartile ranges in parentheses (baseline, skewed distribution) or least square means ± SE (change values). ^a,b^Mean values within a row with unlike superscript letters were significantly different (*P* < 0.05; ANCOVA followed by the least significant difference test). ^A^ANOVA F-test. ^B^ANCOVA with baseline values as a covariate. ^C^Calculated according to the Friedewald formula. Data from participants with TG levels ≥4.52 mmol/L were excluded from the analyses.

### Serum markers of liver injury

The effects of WP and CPs on liver injury markers are given in Table 3. At week 9, supplementation with CPs decreased serum alanine aminotransferase (ALT) and aspartate aminotransferase (AST), whereas participants who received WP or placebo showed no significant changes. After adjusting for baseline, there were significant differences in changes in ALT (*P* = 0.003 and 0.021, respectively) and AST (*P* < 0.001 and =0.014, respectively) in the CPs group compared to the placebo and WP groups. Changes in other biomarkers, including the three serum markers of liver fibrosis hyaluronic acid (HA), type III precollagen (PC III) and type IV collagen (IV-C), did not differ between groups.

**Table 3:** **Effects of treatments on liver injury parameters**

Parameters	Placebo (***n*** = 49)	Whey protein (***n*** = 50)	Corn peptides (***n*** = 47)	***P***value
TB (μmol/L)				
Baseline	13.58 (10.56 - 15.47)	12.12 (10.37 - 14.15)	13.22 (10.94 - 17.33)	0.404^A^
∆9 weeks	0.08 ± 0.34	-0.46 ± 0.34	-0.99 ± 0.35	0.100^B^
ALP (U/L)				
Baseline	65.26 (56.75 - 73.02)	63.64 (53.07 - 75.86)	66.61 (60.30 - 76.29)	0.219^C^
∆9 weeks	-1.09 ± 0.83	-1.05 ± 0.82	-3.23 ± 0.85	0.117^B^
TP (g/L)				
Baseline	64.13 ± 0.60	62.82 ± 0.98	62.00 ± 0.97	0.224^A^
∆9 weeks	0.09 ± 0.52	1.24 ± 0.51	0.13 ± 0.53	0.208^B^
GGT (U/L)				
Baseline	67.44 (56.75 - 84.81)	65.34 (53.67 - 78.78)	68.34 (57.51 - 91.81)	0.361^C^
∆9 weeks	-2.73 ± 1.03	-3.59 ± 1.02	-3.76 ± 1.06	0.753^B^
ALT (U/L)				
Baseline	34.51 (28.96 - 41.48)	32.82 (26.73 - 39.54)	34.47 (28.90 - 44.33)	0.500^C^
Δ9 weeks	0.31 ± 0.67^a^	-0.40 ± 0.66^a^	-2.61 ± 0.68^b^	0.007^B^
AST (U/L)				
Baseline	25.73 (20.42 - 33.24)	25.19 (21.04 - 31.98)	27.46 (22.16 - 35.49)	0.496^C^
∆9 weeks	0.43 ± 0.69^a^	-0.85 ± 0.69^a^	-3.30 ± 0.71^b^	0.001^B^
AST/ALT				
Baseline	0.78 (0.69 - 0.90)	0.80 (0.74 - 0.94)	0.78 (0.66 - 0.87)	0.440^C^
∆9 weeks	-0.02 ± 0.02	0.01 ± 0.02	-0.04 ± 0.02	0.310^B^
HA (ng/mL)				
Baseline	88.69 (69.55 - 98.30)	86.88 (68.66 - 101.78)	82.63 (65.58 - 100.30)	0.677^C^
∆9 weeks	2.78 ± 3.21	1.42 ± 3.19	3.67 ± 3.29	0.884^B^
PC III (ng/mL)				
Baseline	72.11 (62.82 - 85.38)	67.14 (56.86 - 79.45)	75.84 (64.65 - 89.05)	0.079^A^
∆9 weeks	-1.93 ± 1.45	-2.36 ± 1.44	-4.77 ± 1.49	0.344^B^
IV-C (ng/mL)				
Baseline	59.44 (52.78 - 64.22)	58.93 (51.01 - 69.80)	58.60 (52.29 - 70.93)	0.619^C^
∆9 weeks	-1.57 ± 1.26	-0.10 ± 1.24	-2.23 ± 1.28	0.787^B^

TB, total bilirubin; ALP, alkaline phosphatase; TP, total protein; GGT, gamma-glutamyltransferase; ALT, alanine aminotransferase; AST, aspartate aminotransferase; HA, hyaluronic acid; PC-III, type III precollagen; IV-C, type IV collagen.

Values are means ± SE (baseline, normal distribution), medians with interquartile ranges in parentheses (baseline, skewed distribution) or least square means ± SE (change values). ^a,b^Mean values within a row with unlike superscript letters were significantly different (*P* < 0.05; ANCOVA followed by the least significant difference test). ^A^ANOVA F-test. ^B^ANCOVA with baseline values as a covariate. ^C^Kruskal-Wallis H test.

### Serum markers of oxidative stress and inflammation

The effects of WP and CPs on oxidative stress markers superoxide dismutase (SOD), glutathione peroxidase (GPx) and malondialdehyde (MDA) and inflammatory marker tumor necrosis factor-α (TNF-α) are given in Table 4. At week 9, after adjusting for baseline, the CPs group had greater increases in GPx (*P* < 0.001) and SOD (*P* < 0.001) and greater reductions in MDA (*P* < 0.001 and =0.004, respectively) and TNF-α (*P* < 0.001, respectively) compared to the placebo and WP groups. Changes in the four markers did not differ between WP and placebo groups.

**Table 4:** **Effects of treatments on GPx, SOD, MDA and TNF-**α

Parameters	Placebo (***n*** = 49)	Whey protein (***n*** = 50)	Corn peptides (***n*** = 47)	***P***value
GPx (U/mL)				
Baseline	190.48 ± 3.88	184.01 ± 4.79	187.15 ± 4.29	0.571^A^
∆9 weeks	4.86 ± 2.63^a^	9.82 ± 2.60^a^	24.97 ± 2.68^b^	<0.001^B^
SOD (U/mL)				
Baseline	33.78 (28.59 - 36.64)	33.33 (24.88 - 38.55)	29.33 (25.53 - 34.19)	0.065^C^
∆9 weeks	-0.16 ± 0.72^a^	1.42 ± 0.70^a^	7.83 ± 0.73^b^	<0.001^B^
MDA (nmol/mL)				
Baseline	4.70 (4.36 - 5.30)	4.78 (4.24 - 5.30)	4.64 (4.39 - 5.25)	0.992^C^
∆9 weeks	-0.08 ± 0.05^a^	-0.20 ± 0.05^a^	-0.41 ± 0.05^b^	<0.001^B^
TNF-α (pg/mL)				
Baseline	95.85 (78.25 - 110.67)	83.24 (68.58 - 121.55)	104.10 (73.55 - 125.62)	0.228^C^
∆9 weeks	-4.65 ± 2.17^a^	-7.69 ± 2.15^a^	-25.56 ± 2.22^b^	<0.001^B^

GPx, glutathione peroxidase; SOD, superoxide dismutase; MDA, malondialdehyde.

Values are means ± SE (baseline, normal distribution), medians with interquartile ranges in parentheses (baseline, skewed distribution) or least square means ± SE (change values). ^a,b^Mean values within a row with unlike superscript letters were significantly different (*P* < 0.05; ANCOVA followed by the least significant difference test). ^A^ANOVA F-test. ^B^ANCOVA with baseline values as a covariate. ^C^Kruskal-Wallis H test.

### Fatty liver

The effects of WP and CPs on fatty liver are given in Table 5. The proportions of ultrasound grading of fatty liver at baseline and week 9 did not differ between groups. The outcomes of fatty liver in the three groups were similar. No significant treatment effects on fatty liver at study termination were observed in the three groups.

**Table 5:** **Effects of treatments on fatty liver**

	Placebo (***n*** = 49)		Whey protein (***n*** = 50)		Corn peptides (***n*** = 47)	
	Baseline	9-week	Baseline	9-week	Baseline	9-week
Fatty liver grade						
Absent	0 (0)	4 (8.2)	0 (0)	3 (6.0)	0 (0)	4 (8.5)
Mild	39 (79.6)	35 (71.4)	37 (74.0)	34 (68.0)	31 (66.0)	28 (59.6)
Moderate	10 (20.4)	10 (20.4)	13 (26.0)	13 (26.0)	16 (34.0)	15 (31.9)
Outcome						
Improved	5 (10.2)		3 (6.0)		5 (10.6)	
Unchanged	43 (87.8)		47 (94.0)		42 (89.4)	
Deteriorated	1 (2.0)		0 (0)		0 (0)	

Values are numbers with percentages in parentheses. Fatty liver grade (at baseline and 9-week) and outcome did not differ between groups (Kruskal-Wallis H test).

## Discussion

Previous studies have demonstrated that CPs possess various bioactive functions, such as anti-hypertension [23], antioxidant [6, 8–14], improvements in lipid profile [9], facilitation of alcohol metabolism [16, 24, 25], prevention of hepatocellular carcinoma [26], and protection against alcohol- [9, 10] and other hepatotoxic substances-induced liver injury [11, 12], but limited data are available for the effect of CPs in humans. The present randomized trial showed for the first time that CPs supplementation (4 g/d) for 9 weeks had beneficial effects on alcoholic liver injury in men with chronic alcohol consumption and fatty liver, whereas the same dose of WP and placebo did not have these effects.

During alcohol oxidation by alcohol dehydrogenase, hydrogen is transferred from the substrate to nicotinamide adenine dinucleotide (NAD^+^), which converts it to NADH. An increase in the NADH/NAD^+^ ratio favors the deposits of TG in the liver and results in steatosis [27, 28]. Previously, Li et al. observed a reduction in the liver TG levels after CPs supplementation in a mouse model of acute alcoholic liver injury [10]. Zhang et al. observed a reduction in serum TC, LDL-C and HDL-C levels after CPs supplementation in a rat model of early alcoholic liver injury [9]. In this study, supplementation with CPs significantly lowered serum TC and TG after 9 weeks compared to supplementation with WP and placebo. Overall, our data consistently showed that CPs improved the lipid profile. Furthermore, the 19.5% reduction in serum TG level and 8.9% reduction in serum TC level that was observed in this study is likely clinically significant. Therefore, further studies are warranted to confirm the effect of CPs on lipid profile in humans.

CPs might improve the lipid profile by increasing NAD^+^ level. The metabolism of the amino acids, such as Glu, Leu, Ala, and Pro, in CPs may be capable of supplying NAD^+^ and modulating the NADH/NAD^+^ ratio to change the redox system, which is involved in a series of metabolic alterations, including hyperlipemia and fatty liver [24, 27, 28].

Alcohol-induced hepatic damage is characterized by the release of hepatic enzymes into the circulatory system. An elevation of hepatic enzymes in serum indicates cellular leakage and a loss of functional integrity of cell membranes in the liver [29]. Among these enzymes, serum AST and ALT activities are the most commonly used markers of hepatocellular damage/necrosis. Previous studies demonstrated that CPs decreased serum AST and ALT activities in rodent models of alcohol- [9, 30] and other hepatotoxic substances-induced liver injuries [11, 12]. Our results consistently showed that CPs supplementation decreased serum AST and ALT activities in men with chronic alcohol consumption. The hepatoprotective effect of CPs was also confirmed by histopathological examination in rodents: alcohol-induced necrotic hepatocytes, characterized by cell enlargement, nuclear dissolution, microvesicular steatosis with inflammatory and cell infiltration, were ameliorated to some extent by CPs treatment [9, 10].

Alcohol abuse stimulates an overproduction of reactive oxygen species (ROS), lowers cellular antioxidant levels, and leads to hepatic apoptosis (the development of ALD) via oxidative stress mechanism [31]. The precise mechanisms by which CPs protect against alcoholic liver injury are not clear, but previous studies implicated that an antioxidant mechanism might underlie the hepatoprotective effects of CPs [9, 10].

It is well-known that antioxidant enzymes, such as SOD and GPx, provide protection against oxidative stress, but they are easily inactivated by excessive lipid peroxides or other ROS resulting from acute alcohol-induced liver damage [29]. Our results are consistent with previous animal data showing that CPs significantly increased serum activities of SOD and GPx and significantly decreased serum levels of MDA (marker of oxidative stress) [6, 9–12]. The notable antioxidant activities, free radical-scavenging activities and reducing power of CPs were also observed in *in vitro* assay systems by various research groups [6, 8, 14]. It was proposed that the observed antioxidant activity may be due to the presence of some proton-donating peptides in CPs [8]. A recent study suggested that the pentapeptide Gly-His-Lys-Pro-Ser may be the main antioxidant component of CPs [6]. Future work could be directed toward the purification and generation of bioactive peptide sequences.

Inflammation also has an important role in the pathogenesis of ALD [32]. Alcohol can awaken Kupffer cells to be sensitized by lipopolysaccharides and promote the production of TNF-α and ROS. These inflammatory mediators contribute to hepatocyte dysfunction, apoptosis, necrosis, and characteristic fibrosis [31, 32]. In our study, CPs supplementation significantly decreased serum TNF-α levels. This result indicates that an anti-inflammatory mechanism might contribute to the hepatoprotective effect of CPs.

Our study has some limitations. First, only one dose and one time point was investigated in this study. The lack of effects of CPs on fatty liver and other biochemical markers of liver damage is likely due to the relatively low dose used and the relatively short period of treatment. More studies evaluating higher doses for a longer treatment period are needed. In addition, the absence of efficacy of WP is also likely due to the relatively low dose regimen we employed. The results would be more convincing if we had a high-dose whey protein group in the current study. However, the significance of the results relate to the possible superiority of CPs to WP at the same dose with respect to the attenuation of alcoholic liver injury. CPs might have more bioactive peptides that contribute to the hepatoprotective effect than WP. Second, our results pertain to Chinese males with chronic alcohol consumption and mild or moderate fatty liver. Therefore, extrapolation to other populations should be performed with great caution. Further studies are needed to confirm and extend the promising findings in subjects with more severe alcoholic liver injury or other diseases involving oxidative stress.

## Conclusions

In conclusion, the present study is the first report showing that CPs supplementation (4 g/d) for 9 weeks has beneficial effects on lipid profile, oxidative stress and alcoholic liver injury in men with chronic alcohol consumption. The consumption of CPs had beneficial effects on liver injury, and regular CPs supplementation may, at the very least, slow the progression of alcoholic liver injury. Consequently, CPs have the potential to be used as functional foods aimed at the management of ALD in subjects with chronic alcohol consumption.

## Materials and methods

### Participants

A total of 161 men were recruited from Shandong, China through fliers posted in the community and word-of-mouth. The following inclusion criteria were used: age 35–55 years, body mass index (BMI) 20–28 kg/m^2^, alcohol consumption >30 g/d for at least 5 years, and ultrasound-defined fatty liver. Daily alcohol consumption (grams of ethanol per day) was calculated based on the type, quantity, and frequency of alcoholic drinks reported. Participants were not eligible if they had medical conditions, such as cancer, cardiovascular disease, and infection, currently or previously (past 6 months) taking medications and /or dietary supplements known to interfere with liver function, lipid metabolism and inflammatory processes. Participants with pre-existing viral hepatitis and drug-induced liver disease were also excluded. This study was conducted according to the guidelines established in the Declaration of Helsinki, and all procedures involving human participants were approved by the Ethics Committee of the School of Public Health at Fudan University, Shanghai, China. All participants gave written informed consent and received monetary compensation for participation.

### Study design

A 9-week, randomized, double-blind, placebo-controlled study was conducted between September 2011 and August 2012 to assess the hepatoprotective effect of CPs. Potential participants were first screened by phone followed by a secondary screening to perform liver ultrasound examination after an overnight fast. Eligible participants completed a schedule of blood draws, anthropometric measurements and a questionnaire regarding personal health, demographic and alcohol consumption. For practical reasons, the secondary screening was performed on three consecutive days because of the ability to test a limited number of subjects. A total of 161 participants were randomized using a random number generator in Excel software (Microsoft, Seattle, WA) to receive corn starch placebo (*n* = 54), WP (*n* = 54), or identical CPs capsule (*n* = 53) at the same dose of 2 g twice daily. The dose of 4 g/day was adopted based on our previous drinking study, review of previous CPs human studies [24, 25], and the number of capsules so as not to affect compliance with study protocol. Each participant was instructed to maintain his habitual mode of living, including diet, physical activity, and alcohol consumption, during the study. The number of supplements not consumed and log reviews were used to assess compliance. Participants returned at 9 weeks to provide a fasting blood sample and undergo liver ultrasound examination.

### Supplements

CPs powder was obtained by sequential treatment of CGM as previously described [9, 10]. Our previous study showed that the CPs sample was rich in Glu + Gln (22.72 g kg^-1^), Leu (16.73 g kg^-1^), Ala (8.17 g kg^-1^) and Pro (6.52 g kg^-1^), and the average molecular weight of the hydrolyzed peptides was 354 Da, with the majority (96.72%) distributed below 1 kDa (XP,YW, FL, SZ, FY, YL, MC and HG, unpublished data). Whey protein concentrate was purchased from Hilmar Inc. (Hilmar TH 8000, Hilmar, CA, USA). Corn starch placebo was purchased from Lvran Food Co. Ltd. (Xuzhou, Jiangsu, China). All CPs, WP and corn starch were packaged as 0.5 g capsules (Capsugel, Suzhou, Jiangsu, China). All capsules were put into bottles containing 56 capsules (for 1 week) and packaged in color-coded plastic bags containing 4 or 5 bottles (for 4 or 5 weeks) for distribution to participants. An unblinded researcher prepared and masked the samples. When participants finished a 4-bottle package after the first 4 weeks, they were given a second 5-bottle package. Participants were instructed to consume 8 capsules two times daily with breakfast and dinner.

### Anthropometric measurements

Height was measured using a calibrated stadiometer (TZG, Shanghai, China) with 1-mm precision. Body weight was measured on a digital scale (CAMRY EB9005L, Zhongshan, Guangdong, China) in light indoor clothes without shoes. BMI was derived from height and weight measurements.

### Blood measurements

At baseline and week 9, a 12-h fasting morning blood sample was collected, processed and stored at -80°C using a standard protocol. All serum marker concentrations or activities were measured using classical methods and commercial assay kits according to the manufacturers' instructions. Assay kits for TC, TG, and HDL-C were purchased from Beijing BHKT Clinical Reagent Co. Ltd. (Beijing, China). LDL-C concentration was calculated according to the formula of Friedewald: LDL-C (mmol/L) = TC – HDL-C - TG/2.2. Assay kits for ALT, AST, total bilirubin (TB), total protein (TP), gamma-glutamyl transpeptidase (GGT), alkaline phosphatase (ALP), SOD, GPx and MDA were purchased from Nanjing Jiancheng Bioengineering Institute (Nanjing, Jiangsu, China). An ELISA kit for TNF-α was purchased from Cusabio Biotech Co. (Wuhan, Hubei, China). Chemiluminescence assay kits for serum liver fibrosis markers HA, PC III, and IV-C were purchased from Beijing Tigsun Diagnostics Co. Ltd. (Beijing, China). Absorbance was measured using a Bio-Rad 680 microplate reader (Bio-Rad Co., Hercules, CA, USA). Chemiluminescence intensity was monitored on a luminescence reader (FlexStation 3, Molecular Devices, Sunnyvale, CA, USA). Concentrations or activities were calculated based on standard curves. Control samples were run with each assay for quality control purposes. Within- and between-day batch variations, determined as the coefficient of variation, were <10%. Laboratory analyses were performed blind in respect to the assigned treatment.

### Liver ultrasound examination

The diagnosis of fatty liver was based on the results of abdominal ultrasonography (Logic Q700 MR, GE, Milwaukee, WI, USA). Ultrasonography was performed by four experienced radiologists who were blinded to the study according to standardized criteria ((hepatorenal echo contrast, liver brightness, deep attenuation, and vascular blurring) [33]. The presence and severity of fatty liver was recorded using a numbered scoring system (1 = absent; 2 = mild; 3 = moderate; and 4 = severe).

### Sample size and power calculations

A sample size of 53 subjects per group was initially chosen to detect a difference of 5 U/ml SOD (assuming a standard deviation of 8 U/ml) between the placebo and CPs groups with a two-sided α value of 0.05 and a power of 80%, allowing for a 25% dropout.

### Statistical analysis

Data were checked for normality using the Shapiro-Wilk test because the sample size was <50. Data are presented as the means and standard errors for normal variables, medians and interquartile ranges for log-transformed variables (serum TG, TB and PC III) and non-normal variables (age, ALP, GGT, ALT, AST, AST/ALT, HA, IV-C, SOD, MDA, TNF-α), least square means and standard errors for change values, and numbers (*n*) or percentages (%) for categorical variables. For continuous variables, baseline values were analyzed using analysis of variance (ANOVA) or the Kruskal-Wallis H test, and changes from baseline at 9 weeks were compared with analysis of covariance (ANCOVA) with baseline values as a covariate to reduce the effect of differences at baseline. When the overall results were significant, the least significant difference test was used to determine which groups differed. For categorical variables, the Kruskal-Wallis H test or chi-square test were used to evaluate differences between groups. All statistical analyses were performed using the Statistical Package for the Social Sciences 17.0 (SPSS Inc., Chicago, IL, USA). P < 0.05 was considered statistically significant.

## Electronic supplementary material

Additional file 1: Table S1: Compliance of the subjects. (DOCX 13 KB)

## Abbreviations

ALD: Alcoholic liver disease
ALP: Alkaline phosphatase
ALT: Alanine aminotransferase
ANCOVA: Analysis of covariance
ANOVA: Analysis of variance
AST: Aspartate aminotransferase
BMI: Body mass index
CPs: Corn peptides
GGT: Gamma-glutamyl transpeptidase
GPx: Glutathione peroxidase
HA: Hyaluronic acid
HDL-C: High-density lipoprotein cholesterol
IV-C: Type IV collagen
LDL-C: Low-density lipoprotein cholesterol
MDA: Malondialdehyde
PC III: Type III precollagen
SOD: Superoxide dismutase
TG: Triglyceride
TB: Total bilirubin
TC: Total cholesterol
TNF-α: Tumor necrosis factor-α
TP: Total protein
WP: Whey protein.
