# Supplementary material for: Protective effect of corn peptides against alcoholic liver injury in men with chronic alcohol consumption: a randomized double-blind placebo-controlled study
Source: Lipids Health Dis. 2014 Dec 13;13:192. doi: 10.1186/1476-511X-13-192 (PMC4290095; doi:10.1186/1476-511X-13-192)
Supplement: Supplementary file 1 — Additional file 1: Table S1: Compliance of the subjects. (DOCX 13 KB) [file 12944_2014_1188_MOESM1_ESM.docx]

**Additional file**

**Additional file 1**

**Table S1 Compliance of the subjects**

| **Compliance** | **Placebo (*n*=49)** | |  | **Whey protein (*n*=50)** | |  | **Corn peptides (*n*=47)** | |
| --- | --- | --- | --- | --- | --- | --- | --- | --- |
|  | ***n*** | **%** |  | ***n*** | **%** |  | ***n*** | **%** |
| $>$90 | 45 | 91.8 |  | 44 | 88.0 |  | 43 | 91.5 |
| 80-90 | 3 | 6.1 |  | 4 | 8.0 |  | 3 | 6.4 |
| $<$80 | 1 | 2.0 |  | 2 | 4.0 |  | 1 | 2.1 |

Values are expressed as numbers (*n*) of subjects and percentages (%). Compliance did not differ between groups (Kruskal-Wallis H test).
